# Giant mediastinal mass in a 3-year-old boy: A rare presentation of neurofibromatosis type I

**DOI:** 10.22037/ijcn.v15i4.23846

**Published:** 2021

**Authors:** Hosseni KARAMI, Maryam GHASEMI, Amirmasoud TAHERI, Faria ROSTAMKOLAIE, Ali ABBASKHANIAN, Mohammad NADERISORKI

**Affiliations:** 1 Pediatric Hematology & Oncology,Thalassemia Research Center (TRC), Hemoglobinopathy Institute, Mazandaran University of Medical Sciences, Sari, Iran; 2 pathology, Immunogenetics research center,Faculty of medicine, Mazandaran university of medical sciences, Sari, Iran; 3 Medical student,Faculty of Medicine, Mazandaran University of Medical Sciences, Sari, IR Iran; 4 Pediatrics Neurology, Clinical Research Development Unit of Bu-Ali Sina Hospital, Mazandaran university of medical sciences, Sari, Iran

**Keywords:** Superior vena cava syndrome, Mediastinal mass, Neurofibromatosis type 1, Child

## Abstract

Neurofibromatosis type 1 (NF1) is an autosomal dominant disease diagnosed with the presentation of café-au-lait macules, skinfold freckling, iris Lisch nodules, neurofibromas, osseous lesion, and optic gliomas. Mediastinal mass as the first presentation of NF1 is very rare, with a frequency of about 2.7%. Here, we present a rare case of NF1 in a 3-year-old boy admitted with respiratory distress and superior vena cava syndrome.

## Introduction

Neurofibromatosis type 1 (NF1), or Von Recklinghausen disease, is an autosomal dominant disorder caused by mutation in the NF1 gene, located at chromosome 17 (1). This mutation results in diminished or loss of production of a protein named neurofibromin, causing clinical features of this disease (2).

The cardinal signs of this disease are café-au-lait spots, neurofibromas of any type, freckling in the axillae or groin, optic glioma, Lisch nodules, and dysplasia of the sphenoid or long bones. Also, familial history of NF1 in first-degree relatives is another central marker for diagnosis (3).

Orthopedic problems, including scoliosis, osteopenia, and osteoporosis, hypertension, precocious puberty, learning disabilities, such as attention deficit hyperactivity disorder (ADHD), and seldom seizure are the rare presentations or complications of this disease (4). In less than 3% of cases, the disease appears as a primary mediastinal mass (5). Here, we describe a case of NF1 presented by huge mediastinal mass and respiratory distress.

## Case Report

A 3-year-old boy was admitted with severe respiratory distress and tachypnea after upper respiratory tract infection. He had dyspnea, cough, orthopnea, respiratory rate about 43 /min, and mild plethora of the face.

He was the only child of family, and his parents were not relatives. He was born through normal vaginal delivery, and his Apgar score was 10 at birth and 5 minutes after birth. He had no developmental delay, but his weight and height were under 3 percentiles of growth, which was also evident on physical examination. We did not find significant data in his past medical history.

**Figure 1:**
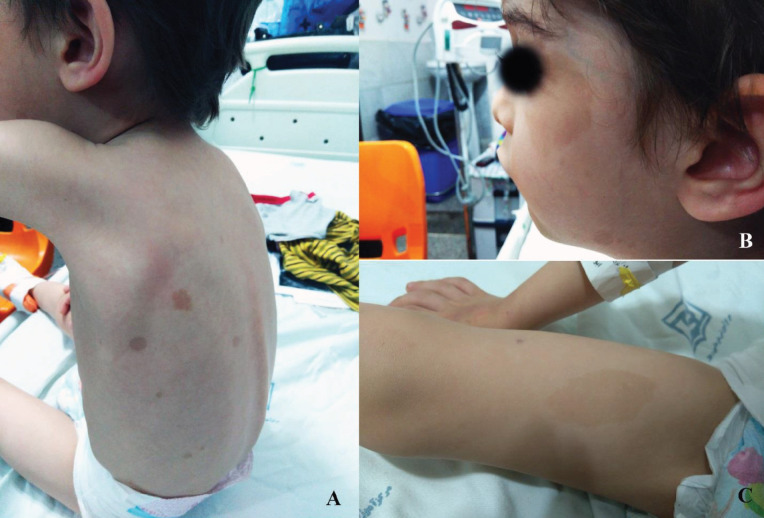
multiple café-au-lait spots on the skin of trunk (1-A), face (1-B), and thigh (1-C)

On physical examination, we found a 5 x 4 cm mass on the left supraclavicular area and neck. The mass was firm and non-tender. Blood pressure was normal. Skin examination revealed multiple café-au-lait spots (Figures 1-A, 1-B, & 1-C).

Laboratory data showed hemoglobin (Hb) 10.9 g/dL, WBC 13300 /µL, neutrophil 29%, eosinophil 7%, monocyte 8%, lymphocyte 56%, platelet count 262000/µL, urea 21mg/dL, creatinine 0.6 mg/dL, uric acid 3.9 mg/dL, and lactate dehydrogenase (LDH) 754 U/L. Beta-HCG was 0.66 m IU/ml and alpha fetoprotein was 1.01 IU/ml.

**Figure 2:**
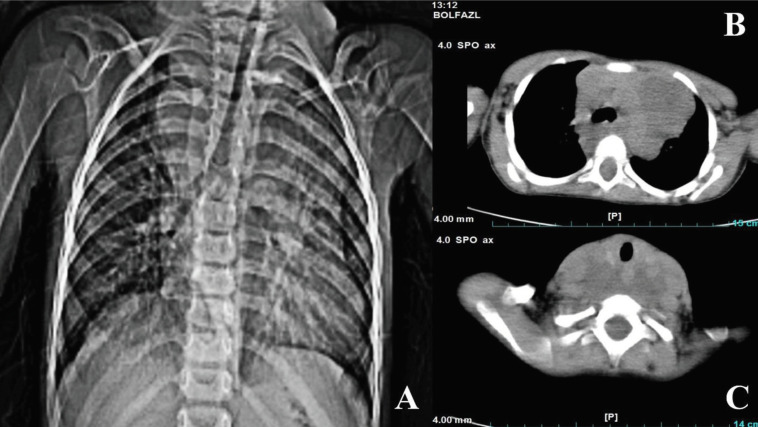
A large mediastinal mass in chest X-ray (2-A) and a heterogenous mass in chest CT scan of thoraces (2-B) with extension to neck (2-C)

Chest radiograph was obtained, which showed a large mediastinal mass (Figure 2-A), and chest CT scan revealed a heterogenous mass on the left mediastinum (Figure 2-B) with extension from thoracic inlet to the neck (Figure 2-C). The mass compressed the neck vessels and airway.

**Figure 3:**
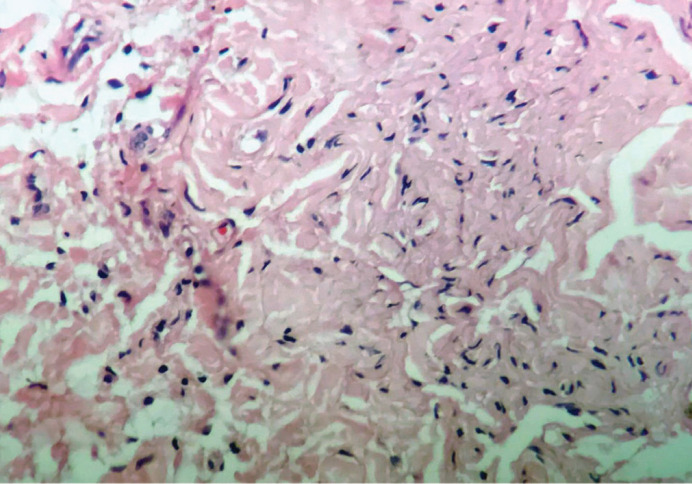
Histologic examination shows spindle cells within wire-like collagen fibrils

Corticosteroids were started because of respiratory distress and superior vena cava syndrome. After three days, the patient became stable, and incisional biopsy was taken from the neck mass, indicating proliferation of spindle cells within wire-like collagen fibrils in loose background, in favor of neurofibromatosis (Figure 3).

## Discussion

Neurofibromatosis type 1 (NF1) is an autosomal dominant disease and about one-half of cases are familial (6). The disease is clinically diagnosed with the presentation of café-au-lait macules, skinfold freckling, iris Lisch nodules, neurofibromas, osseous lesion, and optic gliomas (7). However, all features may not be present in all patients. Mediastinal mass as the first presentation of NF1 is very rare, with a frequency of about 2.7% (5).

Intrathoracic neurofibromatosis mostly arises from ganglia or nerves of sympathetic trunk and rarely from the trachea or esophagus (8, 9). Mediastinal neurofibromas can transform into malignant peripheral nerve sheath tumors (MPNST) (10).

Symptoms and signs of mediastinal neurofibromatosis vary by location, tumor size, and tumor extension to other organs. Superior vena cava syndrome may result due to the impairment of venous return. Dyspnea is a presentation of airway compression or may results from phrenic nerve involvements and hemidiaphragm paralysis (8).

The first imaging modality, chest radiography, shows a homogenous or heterogenous mass, and computed tomography scan technique may detect more details. Magnetic resonance imaging (MRI) in neurofibromatosis type 1 shows areas of T2 hyperintensity, and it seems to be more useful for the diagnosis of NF1-related malignant tumors (11). A sensitivity of 100% and specificity of 77-95% have made PET/CT a superior diagnostic tool for detecting malignant transformation in NF1 (12). 

Elongated spindle-shaped cells and pleomorphic fibroblast-like cells are the main histologic findings in NF1 (13). Molecular testing is now available for the diagnosis of NF1, but it is not routinely indicated for clinical care, especially because of less genotype-phenotype correlations (14, 15). Nevertheless, it may be helpful and is recommended in young children with multiple café-au-lait spots only and no family history (16).

Finally, other differential diagnoses must be considered in any patient with signs and symptoms similar to NF1, like NF1-like syndrome, familial café-au lait spots (FCALS), and segmental NF1 (17). Our patient was a rare case of mediastinal NF1, which presented by respiratory distress and superior vena cava syndrome. Base on clinical manifestations and histologic findings, our patient was rapidly referred to a thoracic surgeon. Surgery is the main treatment approach, and it is recommended that surgery be performed in one step (18).

## In Conclusion, 

Despite the rarity of NF1 as a mediastinal mass, this diagnosis should be considered as a differential diagnosis, especially when one or more other signs and symptoms are present.
